# “A real-world evidence” in reduction of volatile anesthetics by BIS-guided anesthesia

**DOI:** 10.1038/s41598-020-68193-x

**Published:** 2020-07-09

**Authors:** Yan-Yuen Poon, Han-Chen Chang, Min-Hsien Chiang, Kuo-Chuan Hung, Hsiao-Feng Lu, Chih-Hsien Wang, Jo-Chi Chin, Shao-Chun Wu

**Affiliations:** 1 Department of Anesthesiology, Kaohsiung Chang Gung Memorial Hospital, Chang Gung University College of Medicine, No. 123, Ta-Pei Rd., Niao-Song Dist., Kaohsiung City, 833 Taiwan; 2 Department of Anesthesiology, Chi Mei Medical Center, No.901, Zhonghua Rd. Yongkang Dist., Tainan City, 710 Taiwan

**Keywords:** Health care, Medical research, Neurology

## Abstract

Many well-controlled clinical studies have shown that BIS-guided anesthesia could prevent intraoperative awareness and improve postoperative morbidity and mortality, by optimizing the amount of volatile anesthetics administered to patients. However, we questioned if the previously reported advantages of BIS-guided anesthesia in controlled studies would still apply in real-world settings. This retrospective study based on real-world settings clarified the role of BIS-guided anesthesia in reducing anesthetic consumption. We obtained anesthesia records from an electronic database of a medical center in southern Taiwan. A total of 6,713 cases were enrolled, where 1,324 cases receiving sevoflurane underwent BIS-guided anesthesia and 378 received desflurane; further, 3,819 receiving sevoflurane underwent standard anesthesia practice and 1,192 cases received desflurane. The median (25–75% interquartile values) of the average hourly consumption of sevoflurane or desflurane decreased significantly under BIS-guided anesthesia [10.5 (8.7–13.0) mL/h and 17.4 (13.7–21.1) mL/h, respectively] compared to that under standard anesthesia practice [11.4 (9.0–14.5) mL/h, and 20.2 (15.8–25.0), mL/h, respectively]. Furthermore, the average hourly consumption of these two volatile anesthetics varied inversely with age and anesthesia time in both groups. A significant reduction was found in the hourly consumption of volatile anesthetics in patients under BIS-guided anesthesia compared to standard anesthesia practice in different age groups or different anesthesia time. We concluded that BIS-guided anesthesia could reduce consumption of volatile anesthetics in real-world settings as well.

## Introduction

Bispectral index monitor (BIS) is an anesthesia depth measurement tool that has gained clinical popularity in recent years after many clinical trials supported its use in lowering the risk of intraoperative awareness, shortening recovery time, and improving postoperative morbidity and mortality. In these studies, BIS reduced the total use of anesthetic agents, reducing the total anesthesia cost compared with standard anesthesia practice^1^. Consumption of volatile anesthetics in BIS-guided anesthesia ensures the appropriate amount given and, more importantly, prevents intraoperative awareness. Conventional, well-controlled clinical trials utilized different tools to estimate volatile anesthetic consumption, and most results point to reduced anesthetic consumption in BIS-guided anesthesia^2–6^. These traditional clinical trials were conducted with a specific study population, in specialized environments, and adhere to a list of eligibility criteria to control variability and ensure the quality of the data^7^. However, in practice, restrictions or patient selections would not be ethically acceptable. The impetus for this study comes from the notion that conventional studies do not always reflect the real-world settings. With modern, sophisticated anesthesia machines available that calculate volatile anesthetics consumed during each anesthesia and the accessibility to electronic anesthesia records, we can identify the inter-relationships of volatile anesthetics consumed in each anesthesia versus various independent variables under real-world settings. Distinct from previous well-controlled clinical trials that investigated the influence of BIS-guided general anesthesia on intraoperative minimal alveolar concentration (MAC)^1^, we investigated the role of BIS-guided general anesthesia on the reduction of volatile anesthetics based on a real-world setting.

## Results

**Figure 1:**
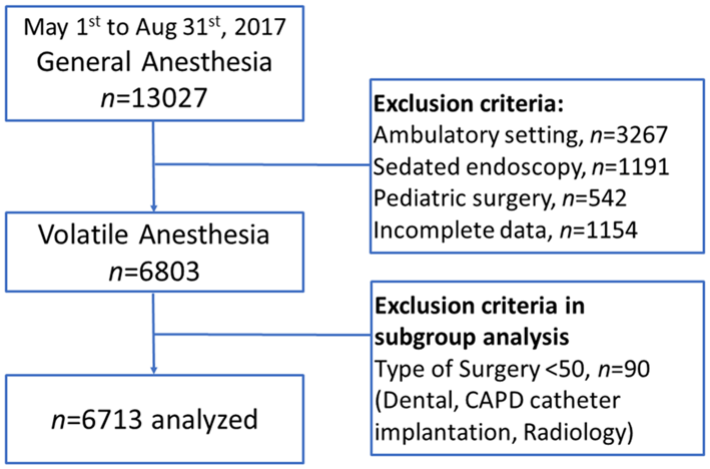
Flow diagram of Adult Volatile Anesthesia analyzed in this study.

A total of 13,027 general anesthesia records from May 1st, 2017 to Aug 31st, 2017, were retrieved from our hospital’s electronic database. We recruited 6,803 cases after excluding those with ambulatory surgeries, sedated endoscopy, pediatric surgery, and those with missing data. Table 1 summarizes the demographic characteristics of the cases. Cases were stratified into five different categories: anesthesia time, age, gender, ASA physical status, and surgical entities. For statistical purposes, infrequently performed surgeries during the study period were excluded from this study. These included dental surgery (n = 49), CAPD catheter implantation (n = 32), and radiological procedures (n = 9) (Table 1). Finally, a total of 6,713 cases of volatile general anesthesia were included for statistical analysis (Fig. 1).

**Table 1:** Demographic characteristics of (N = 6,803) in adult hospitalized surgery from May 1st, 2017 to Aug 31st, 2017.

	**Total case numbers n (%)**	**BIS n (%)**	**Standard practice n (%)**
Anesthesia time			
< 2 h	1,620 (23.8%)	276 (16.1%)	1,344 (26.4%)
2–4 h	3,205 (47.1%)	746 (43.6%)	2,459 (48.3%)
4–6 h	1,209 (17.8%)	372 (21.7%)	837 (16.4%)
6–8 h	424 (6.2%)	175 (10.2%)	249 (4.9%)
≧8–10 h	345 (5.1%)	143 (8.4%)	202 (4.0%)
Age (years old)			
21–40	1,279 (18.8%)	224 (13.1%)	1,055 (20.7%)
41–60	2,615 (38.4%)	634 (37.0%)	1981 (38.9%)
61–80	2,622 (38.5%)	768 (44.9%)	1854 (36.4%)
> 80	287 (4.2%)	86 (5.0%)	201 (3.9%)
Gender (M/F)			
Male	3,413 (50.2%)	743 (43.4%)	2,421 (47.6%)
Female	3,390 (49.8%)	969 (56.6%)	2,670 (52.4%)
Weight (kg)	65.25 ± 13.25	64.94 ± 13.79	66.47 ± 22.19
ASA physical status			
1	370 (5.4%)	86 (5.0%)	284 (5.6%)
2	3,880 (57.1%)	978 (57.2%)	2,902 (57.0%)
3	2,419 (35.6%)	636 (37.2%)	1783 (35.0%)
4	124 (1.8%)	10 (0.6%)	114 (2.2%)
5	7 (0.1%)	0 (0.0%)	7 (0.1%)
Surgery			
Cardiothoracic vascular	299 (4.4%)	134 (7.8%)	165 (3.2%)
Otolaryngology	510 (7.5%)	78 (4.6%)	432 (8.5%)
General	1,167 (17.2%)	511 (29.8%)	656 (12.9%)
Urology	632 (9.3%)	157 (9.2%)	475 (9.3%)
Gynecology	521 (7.7%)	178 (10.4%)	343 (6.7%)
Neurosurgery	564 (8.3%)	114 (6.7%)	450 (8.8%)
Ophthalmology	219 (3.2%)	29 (1.7%)	190 (3.7%)
Orthopedics	1517 (22.3%)	246 (14.4%)	1,271 (25.0%)
Plastic reconstructive	556 (8.2%)	22 (1.3%)	534 (10.5%)
Colo-rectal	354 (5.2%)	166 (9.7%)	188 (3.7%)
Trauma	374 (5.5%)	67 (3.9%)	307 (6.0%)
Dental surgery	49 (0.7%)	0 (0.0%)	49 (1.0%)
CAPD catheter implantation	32 (0.5%)	9 (0.5%)	23 (0.5%)
Radiology	9 (0.1%)	1 (0.1%)	8 (0.2%)

**Figure 2:**
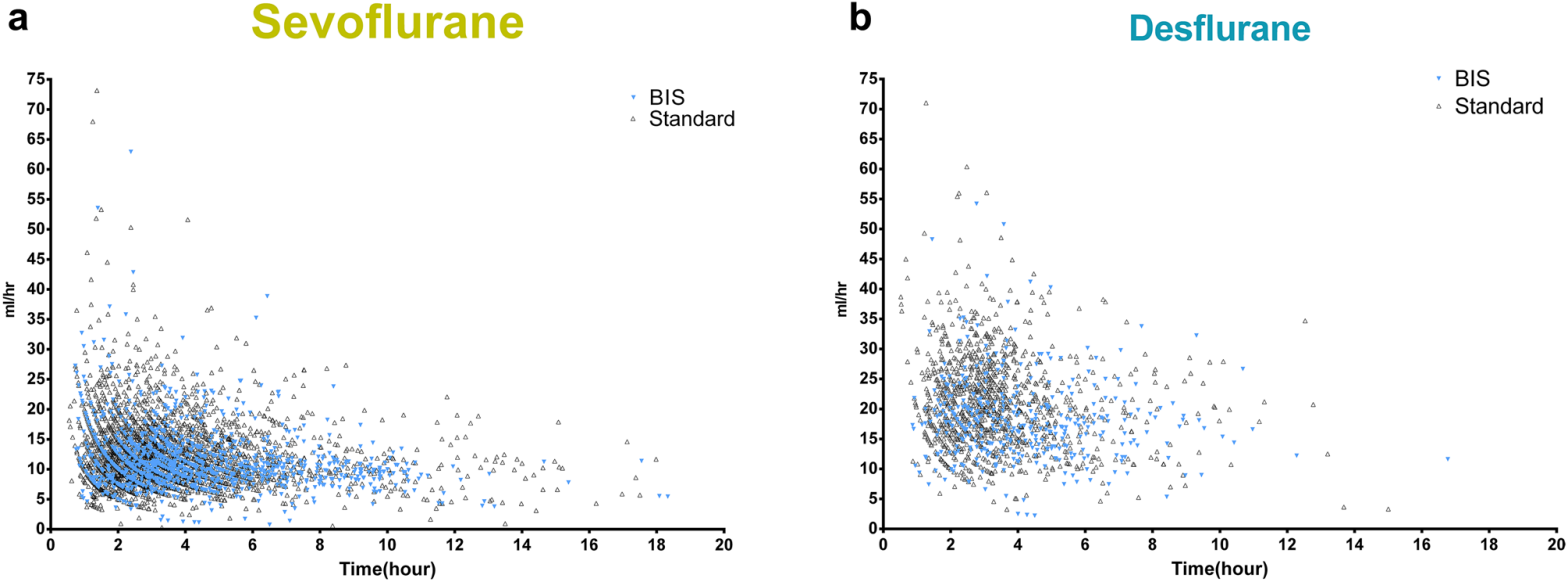
Indicate hourly consumption of sevoflurane (**a**) or desflurane (**b**) versus anesthesia time in BIS guided and Standard practice group.

The general relationship between the hourly consumption of volatile anesthetics and anesthesia times were shown in Fig. 2a,b, respectively. A series of parallel counter curves illustrate a decrease in hourly consumption of volatile anesthetics versus anesthesia time in both sevoflurane (Fig. 2a) and desflurane (Fig. 2b) irrespective of anesthesia practice. The median (25–75% interquartile values) of the hourly consumption of sevoflurane and desflurane in all surgeries (Table 2) under BIS-guide anesthesia were 10.5 (8.7–13.0) mL/h and 17.4 (13.7–21.1) mL/h respectively which were significantly lesser than the corresponding hourly consumptions under standard anesthesia practice [11.4 (9.0–14.5) mL/h, and 20.2 (15.8–25.0), mL/h, respectively].

**Table 2:** Consumption of volatile anesthetic guided by BIS or standard practice by anesthesia time.

**Group**	**BIS (mL/h)**	**Standard practice (mL/h)**	***P***
Sevoflurane (median [25–75% interquartile values])			
	10.5 (8.7–13.0), n = 1,324	11.4(9.0–14.5), n = 3,819	< 0.001
Desflurane (median [25–75% interquartile values])			
	17.4(13.7–21.1), n = 378	20.2(15.8–25.0), n = 1,192	< 0.001
Sevoflurane (mean [95% CI])			
2 h	11.5(11.2–11.7) , n = 1,324	12.3(12.2–12.5) , n = 3,819	< 0.001
4 h	11.2(10.9–11.4) , n = 1,102	12.0(11.94–12.2) , n = 2,759	< 0.001
6 h	10.6(10.2–10.9) , n = 529	11.7(11.4–12.0) , n = 992	< 0.001
8 h	10.1(9.6–10.6) , n = 255	11.0(10.5–11.5) , n = 347	0.016
≥ 10 h	9.4(8.9–9.9) , n = 123	10.0(9.3–10.8) , n = 155	0.160
Desflurane (mean [95% CI])			
2 h	18.3(17.6–19.0), n = 378	20.9(20.5–21.4), n = 1,192	< 0.001
4 h	18.2(17.4–19.0), n = 332	20.8(20.3–21.3) , n = 944	< 0.001
6 h	17.2(16.2–18.2) , n = 161	19.0(18.1–19.8) , n = 279	0.009
8 h	17.5(15.9–19.1) , n = 63	18.0(16.5–19.6) , n = 91	0.643
≥ 10 h	16.4(13.5–19.2) , n = 20	17.9(15.3–20.5) , n = 36	0.433

Data were expressed as median (25–75% interquartile values) or mean (95% CI).

**Figure 3:**
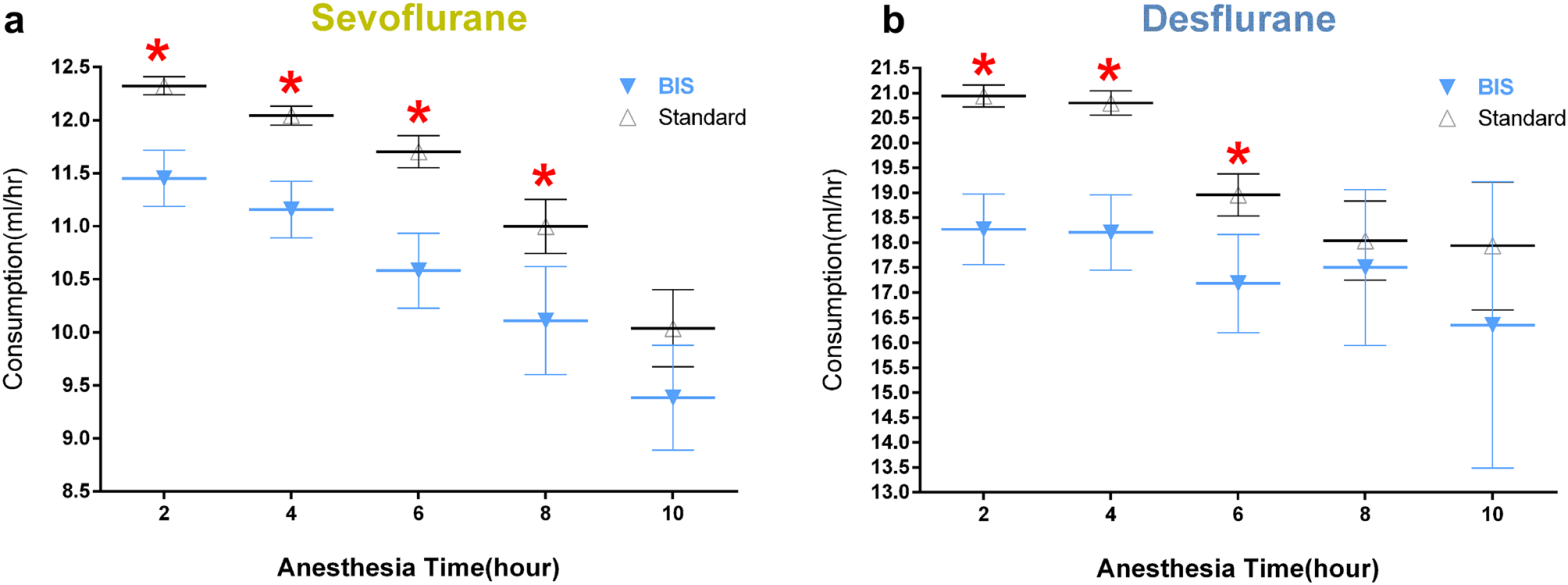
Indicate hourly consumption of BIS guided and Standard practice group in sevoflurane (**a**) or desflurane (**b**) in 5 groups of anesthesia time.

We also evaluated the effect of anesthesia time on the consumption of volatile anesthetics. Patients with different anesthesia time were divided into five groups for comparisons. The hourly consumption of sevoflurane was significantly less in 2 h, 4 h, 6 h, and 8 h anesthesia times under BIS-guided anesthesia as compared with the corresponding anesthesia times under standard anesthesia practice (Table 2, Fig. 3a). Similarly, the hourly consumption of desflurane was significantly lesser in 2 h, 4 h, 6 h anesthesia time under BIS-guided anesthesia than the corresponding anesthesia times under standard anesthesia practice (Table 2, Fig. 3b).

**Figure 4:**
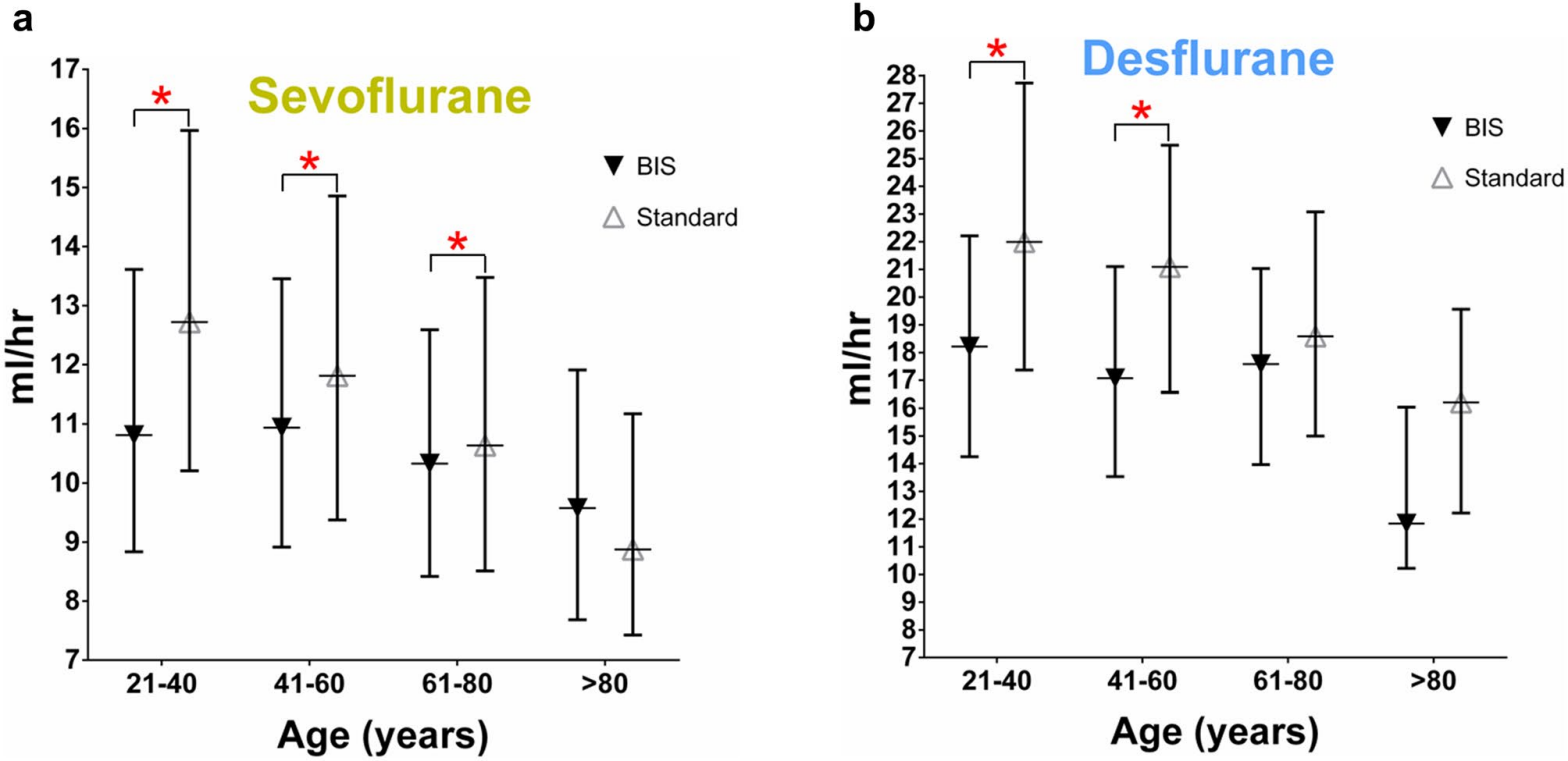
Hourly consumption of BIS-guided and Standard practice group in sevoflurane (**a**) or desflurane (**b**) volatile anesthetics in 4 groups of age.

We then evaluated the effect BIS-guided anesthesia had on reducing volatile anesthetics among different age groups. The hourly consumption of sevoflurane was significantly lower in BIS-guided anesthesia than in standard anesthesia practice in the 21–40, 41–60, and 61–80 year-old groups, as shown in Table 3 and Fig. 4a. The hourly consumption of desflurane was significantly lower in BIS-guided anesthesia than in standard anesthesia practice for the 21–40 and 41–60 year-old groups (Table 3 and Fig. 4b).

**Table 3:** Consumption of volatile anesthetic guided by BIS or Blood pressure by age.

**Age (years old)**	**BIS (mL/h)**	**Standard practice (mL/h)**	***P***
Sevoflurane			
21–40	10.8 (8.8–13.6) (n = 152)	12.7 (10.2–16.0) (n = 741)	< 0.001
41–60	10.9 (8.9–13.5) (n = 483)	11.8 (9.4–14.9) (n = 1,491)	< 0.001
61–80	10.3 (8.4–12.6) (n = 616)	10.6 (8.5–13.5) (n = 1,429)	0.032
> 80	9.6 (7.7–11.9) (n = 73)	8.9 (7.4–11.2) (n = 158)	0.461
Desflurane			
21–40	18.2 (14.3–22.2) (n = 70)	22.0 (17.4–27.7) (n = 299)	< 0.001
41–60	17.1 (13.5–21.1)(n = 147)	21.1 (16.6–25.5) (n = 447)	< 0.001
61–80	17.6 (14.0–21.0)(n = 148)	18.6 (15.0–23.1) (n = 403)	0.095
> 80	11.8 (10.2–16.0) (n = 13)	16.2 (12.2–19.6) (n = 43)	0.053

Data were expressed as median (25%-75% interquartile values).

Gender effects on the hourly consumption of volatile anesthetics in BIS-guided anesthesia and standard anesthesia practice were evaluated. In BIS-guided anesthesia and standard anesthesia practice, the hourly consumption of sevoflurane was significantly higher in male patients than in female patients. The hourly consumption of desflurane was significantly lower in female patients in standard anesthesia practice (Table 4).

**Table 4:** Consumption of volatile anesthetic guided by BIS or Standard practice by gender.

	**Male**	**Female**	***P***
Sevoflurane			
BIS (mL/h)	10.8 (9.0–13.6) (n = 596)	10.1 (8.5–12.5) (n = 728)	0.001
Standard practice (mL/h)	11.9(9.4–15.2) (n = 2019)	10.8(8.7–13.6) (n = 1,800)	< 0.001
Desflurane			
BIS (mL/h)	17.7 (13.9–21.8)(n = 142)	17.2 (13.4–20.9) (n = 236)	0.437
Standard practice (mL/h)	22.1 (17.0–27.7) (n = 599)	19.0 (15.1–22.8) (n = 593)	< 0.001

Data were expressed as median (25–75% interquartile values).

Postoperative events in BIS and Standard practice groups was shown in Table 5. The occurrence of intraoperative awareness was 0.1% in BIS group and 0.1% in standard anesthesia group without significant difference. The occurrence of postoperative headache or dizziness was 1.8% in BIS group and 1.4% in standard anesthesia group without significant difference. The occurrence of postoperative nausea and vomiting was 12.4% in BIS group and 14.1% in standard anesthesia group without significant difference. The occurrence of postoperative respiratory event was 2.4% in BIS group and 1.7% in standard anesthesia group without significant difference. The occurrence of cerebrovascular event was 0.2% in BIS group and 0.2% in standard anesthesia group without significant difference. Patients’ satisfaction showed no significant difference between two groups.

**Table 5:** Postoperative events in BIS and Standard practice groups.

	**BIS n (%)**	**Standard practice n (%)**	***p***
Intraoperative awareness			0.663
No	1,700 (99.9%)	5,007 (99.9%)	
Yes	2 (0.1%)	4 (0.1%)	
Headache/dizziness			0.191
No	1671 (98.2%)	4,942 (98.6%)	
Yes	31 (1.8%)	69 (1.4%)	
Postoperative nausea/vomiting			0.076
No	1,491 (87.6%)	4,304 (85.9%)	
Yes	211 (12.4%)	707 (14.1%)	
Respiratory event			0.061
No	1661 (97.6%)	4,926 (98.3%)	
Yes	41 (2.4%)	85 (1.7%)	
Cerebrovascular event			0.785
No	1698 (99.8%)	5,001 (99.8%)	
Yes	4 (0.2%)	10 (0.2%)	
Patients’ satisfaction	4.0 (4.0–5.0)*	4.0 (4.0–5.0)*	0.077

*Data were expressed as median (25–75% interquartile values).

## Discussion

Since the introduction of BIS monitor to anesthesia in 1994^8^, numerous clinical trials have been carried out to evaluate its efficacy in preventing intraoperative awareness^1,9–11^, lowering postoperative morbidity^12,13^ and mortality^14,15^, enhancing early postoperative recovery^16,17^, and reducing intraoperative consumption of volatile anesthetics^5,17–19^. It is reasonable to speculate that more than adequate consumption of volatile anesthetics may increase the risk of unwanted postoperative side effects. Many well-controlled clinical trials supported the role of BIS-guided anesthesia in maintaining an adequate level of anesthesia without an overdose of volatile anesthetics. However, experiences in the real-world may differ from well-controlled settings; results from well-controlled studies may have interpretation bias in real-world settings. The results support the role of BIS-guided anesthesia on conserving volatile anesthetics consumption based on real-world evidence. The meta-analysis by Gao et al.^20^ showed that BIS monitoring has no obviously advantage in decreasing perioperative awareness incidence in volatile anesthesia. The occurrence of intraoperative awareness in our results has the same consequent.

One of the interesting findings revealed in this study was a series of parallel counter curves either in BIS-guided anesthesia or in standard anesthesia practice shown in the scatter diagrams (Fig. 2a,b). These parallel counter curves resembled the pharmacokinetic profiles of volatile anesthetics. High hourly consumptions of both volatile anesthetics were recorded in most surgeries that lasted less than 1 h, which reflected the early phase of uptake of volatile anesthetics when high concentration gradient existed between alveolar and exogenous gas supply. A “genuflection” followed in surgeries of 2-h duration, which marked the transition from the active uptake phase to an equilibrium state. In surgeries lasting more than 2 h, the hourly consumption became fairly constant, and it denoted that an equilibrium state was attained. The physicochemical properties of these two volatile anesthetics are different that their rates of uptake should be reflected in the scatter diagrams (Fig. 2a,b); however, more data, especially on desflurane (n = 1,570 in desflurane vs. n = 5,143 in sevoflurane), are needed before a complete pharmacokinetic profile could be constructed for comparison. The hourly consumption of these two volatile anesthetics decreased as the anesthesia times increased (Fig. 3a,b), and the consumption of these two volatile anesthetics was significantly lesser under BIS-guided anesthesia than under standard anesthesia practice. These results are in accordance with previous clinical studies showing declining consumption of volatile anesthetics as anesthesia time increased. Most pharmacokinetic profiles of volatile anesthetics can only be investigated in sophisticated laboratories or under well-controlled clinical conditions; however, this study may provide an example of utilizing clinical data to establish pharmacokinetic profiles of volatile anesthetics under real-world settings.

Another interesting finding revealed by these parallel counter curves in the scatter diagrams (Fig. 2a,b) was the layout of these counter curves. Further analysis showed that top layer curves represented younger age groups, while lower layer curves represented older age groups. The hourly consumption of the two volatile anesthetics was decreased in elderly patients compared to that in younger patients (Fig. 4a,b). This result is in agreement with the results of previous clinical studies showing less uptake of volatile anesthetics in elderly patients^21^.

Different reports utilize various ways to estimate the consumption of volatile anesthetics. Punjasawadwong et al. in his review article reported BIS-guided anesthesia reduced the requirement for volatile anesthetics (desflurane, sevoflurane, isoflurane) by 0.65 standardized mean difference of minimal alveolar concentration equivalents in 985 patients^1^. Aime et al. reported BIS-guided anesthesia required 29% less sevoflurane in 140 adult patients; the consumption was estimated by weighing sevoflurane vaporizers before and after anesthesia^18^. Basar et al. reported BIS-guided anesthesia reduced sevoflurane usage by 4.37% in 60 adult patients, the consumption of which was estimated by recording the end-tidal sevoflurane concentration every 5 min during surgery, and computed at the conclusion of surgery^19^. Boztug et al. reported BIS-guided anesthesia reduced the maintenance anesthetic concentration by 25% in 50 adult patients^2^. Kreuer et al. reported BIS-guided anesthesia reduced desflurane consumption by 6.6% in 120 adult patients. The consumption was estimated by weighing sevoflurane vaporizers before and after anesthesia^22^. Modern anesthesia machines automatically report the total consumption of volatile anesthetics at the conclusion of each anesthesia, making an accurate estimation of consumption possible in this study. This study provided a more conceivable concept on BIS-guided anesthesia in the reduction of volatile anesthetics consumption than previous studies.

There are some limitations in our study. First, this is a retrospective study, the interpretation of results would be interfered by potential bias. Second, the study focuses on the benefit of volume consumption by BIS-guided general anesthesia. The beneficial of BIS on patients’ safety such as intraoperative vital signs were not investigated. Third, we did not discuss the relation between the decreased volatile anesthetics consumption and the cost reduction. In conclusion, well-controlled clinical studies remain the basis for testing any new drugs or technologies that are to be introduced into real-world practice. Although our study provided real-world evidence to support the role of BIS-guided anesthesia in providing an adequate anesthesia level, more clinical evidence is needed to justify the advantages inherent to BIS-guided anesthesia.

## Methods

Before beginning, the study was approved by the Institutional Review Board of Kaohsiung Chang Gung Memorial Hospital (IRB number: 201800207B0). Informed consent was waived because of the retrospective nature of the study. All methods were performed in accordance with the relevant guidelines and regulations. We retrieved anesthesia records from May 1st, 2017 to Aug 31st, 2017, from the hospital’s database, and a total of 13,027 general anesthesia records were collected. Exclusion criteria included ambulatory surgeries, sedated endoscopy, pediatric surgeries, a limited number of uncommon procedures (case numbers less than 50), and any record with missing data. Recruited patients received either sevoflurane or desflurane as their primary general anesthetic. The general anesthesia was carried out in semiclosed circuit with fresh gas flow of 1 L/min. The anesthesia machines automatically record the consumption of volatile anesthetics at the conclusion of anesthesia. Hourly consumption was calculated depending on the volatile anesthetics consumption and the total anesthesia time. Postoperative daily visit was performed by the well-trained nurse anesthetists and the postoperative events within 72 h including intraoperative awareness, headache or dizziness, postoperative nausea and vomiting, respiratory event, cerebrovascular event, and patients’ satisfaction were recorded.

We divided patients into two major groups as the BIS-guided anesthesia group and the standard anesthesia practice group, and these two groups formed the basis for comparing the consumption of volatile anesthetics in different patients. To elucidate the effect of anesthesia time in these two major groups, we allocated patients according to anesthesia time, namely 2-h, 4-h, 6-h, 8-h, and 10-h. To elucidate the effect of age on the consumption of volatile anesthetics in these two major groups, we stratified patients into four age groups, 21–40 years, 41–60 years, 61–80 years, and over 80 years for comparison.

As a standard practice in our hospital, general anesthesia was induced with propofol (1 to 2 mg/kg). The use of rocuronium (1 mg/kg) or *cis*-atracurium (0.2 mg/kg), fentanyl (1 mcg/kg) or alfentanil (10 mcg/kg), desflurane (1 to 1.3 MAC) or sevoflurane (1 to 1.3 MAC) depends on the anesthesiologists’ preferences. Nitrous oxide, midazolam or other amnestic drugs except propofol was not used in induction and maintenance of general anesthesia in our study. The patient decided whether or not to utilize BIS-guided anesthesia. In the BIS-guided group, the BIS score was kept in the range of 40 to 60 during anesthesia. In the standard anesthesia practice group, volatile anesthetics were titrated against blood pressure and heart rate changes during anesthesia to maintain stable blood pressure and heart rate within 20% of the patient’s normal range. A fresh gas flow of 50% oxygen with air was kept to 1 L/min. Maintenance of neuromuscular blocking agents or opioids depended on surgical stimulus, anesthesiologists’ preferences, and objective vital signs (more than 20% increase in heart rate, systolic blood pressure and mean arterial pressure). The total consumption of volatile anesthetics was automatically recorded by the anesthesia machine Avance (GE Datex-Ohmeda, Madison, WI), S/5 ADU(GE Datex-Ohmeda, Madison, WI), Carestation 620 (GE Datex-Ohmeda, Madison, WI), or Primus (Drägerwerk, AG, Lübeck, Germany).

### Statistical analysis

We used the Kolmogorov–Smirnov test to address the normality of continuous variables. Anesthetic consumption (mL/h) were compared by Student’s *t*-test with normal distribution and expressed as mean (95% confidence interval, CI). Non-normally distributed data distribution were expressed as median (25–75% interquartile values) and tested with Mann–Whitney U test. We performed statistical analysis with SPSS version 22.0 (IBM Corp., Armonk, NY, USA) in this study. A *P* < 0.05 was considered statistically significant.
